# Do joint-preserving hip procedures compromise subsequent total hip arthroplasty? A meta-analysis of complications, functional outcome and survivorship

**DOI:** 10.1051/sicotj/2024018

**Published:** 2024-06-07

**Authors:** En Lin Goh, Oliver R. Boughton, Thomas Donnelly, Colin G. Murphy, James Cashman, Connor Green

**Affiliations:** 1 Oxford Trauma and Emergency Care, Nuffield Department of Orthopedics Rheumatology and Musculoskeletal Sciences, Kadoorie Centre, University of Oxford Oxford OX3 9DU United Kingdom; 2 National Orthopedic Hospital Cappagh Cappagh Road, Cappoge Dublin 11 D11 EV29 Ireland; 3 Department of Trauma and Orthopedics, Merlin Park Hospital EC5, Old Dublin Road Galway Ireland; 4 University College Dublin, School of Medicine Belfield Dublin 4 Ireland

**Keywords:** Osteotomy, Femoral, Acetabular, Hip preserving, Total hip arthroplasty

## Abstract

*Background*: Joint-preserving hip operations can help relieve pain and delay the need for long-term joint arthroplasty. Previous research has not identified procedures that can compromise outcomes following total hip arthroplasty (THA). This meta-analysis aims to evaluate the effect of joint-preserving hip operations on outcomes following subsequent THA. *Methods*: MEDLINE, EMBASE and Scopus databases were searched from the date of inception until February 2024. All studies comparing outcomes following THA in individuals with (PS) and without prior surgery (NPS) of the femur or pelvis were included. Data on operative time, blood loss, intra- and post-operative complications, functional outcomes, and implant survivorship were extracted. *Results*: 16 studies, comprising 2576 patients were included (PS = 939, NPS = 1637). The PS group was associated with significantly longer operative time [MD: 8.1, 95% CI: 4.6–11.6], significantly greater blood loss [MD: 167.8, 95% CI: 135.6–200.0], and a higher risk of intra-operative peri-prosthetic fracture [RR: 1.9, 95% CI: 1.2–3.0], specifically, with prior femoral osteotomy. There were no differences in terms of risks of dislocation [RR: 1.8, 95% CI: 1.0–3.2], implant loosening [RR: 1.0, 95% CI: 0.7–1.5], or revision surgery [RR: 1.3, 95% CI: 1.0–1.7] between the two groups. The PS group was associated with significantly poorer improvements in functional outcome [MD: −5.6, 95% CI: −7.6–(−3.5)], specifically, with prior acetabular osteotomy. Implant survivorship in the two groups was comparable after one year [HR: 1.9, 95% CI: 0.6–6.2] but significantly inferior in the PS group after five years [HR: 2.5, 95% CI: 1.4–4.7], specifically, with prior femoral osteotomy. *Conclusion*: Joint-preserving hip operations are associated with greater intra-operative challenges and complications. In subsequent joint arthroplasty, prior acetabular procedures affect functional outcomes while prior femoral procedures influence implant survivorship. Hip pain due to the morphological sequelae of pediatric hip pathology can be debilitating at a young age. Surgical decision-making at that time needs to consider the survivorship of a THA implanted at that young age against the consequences of hip preservation surgery on further THA.

## Introduction

Joint-preserving hip procedures encompass a variety of operations including arthroscopy, acetabular and femoral osteotomies, fibular grafts, and fusion [1, 2]. The principles of treatment are to relieve pain and improve function, thus, delaying the need for long-term total hip arthroplasty (THA) [1, 3, 4]. These procedures are used in managing the sequelae of childhood disorders such as developmental dysplasia of the hip (DDH), Perthes disease, and slipped capital femoral epiphysis (SCFE) [1].

THA in patients with prior acetabular and femoral osteotomies can be more technically challenging. However, data on clinical and functional outcomes following THA in this population has yielded mixed results, which can be attributed to poor study quality, bias and confounding influences, and the absence of long-term follow-up [3]. It is generally acknowledged that femoral osteotomies involving realignment of the proximal femoral canal make arthroplasty more challenging, but it is unclear whether complications are significantly increased in hip arthroplasty as a result of prior femoral or acetabular surgery.

There is a clear clinical need to understand how prior acetabular or femoral osteotomies can influence outcomes following THA in this patient population. The present meta-analysis aims to evaluate the effect of joint-preserving hip procedures on outcomes following subsequent THA.

## Material and methods

Literature search methods, inclusion and exclusion criteria, outcome measures and statistical analysis were defined according to the Meta-analysis Of Observational Studies in Epidemiology (MOOSE) [5]. Patients were not involved in the conception, design, analysis, drafting, interpretation or revision of this research. Thus, ethics approval was not required.

## Search strategy

The following databases were searched, the last search on 14th February 2024: (a) MEDLINE (1946 until May 2023) via OvidSP; (b) MEDLINE in-process and other non-indexed citations (latest issue) via OvidSP; (c) Ovid EMBASE (1974 to latest issue); (d) Scopus (1996 till present). Three search strings were used, which were then linked by the AND modifier. First search string: “pelvis” OR “hip” OR “femur” OR “acetabulum”; second search string: “total hip arthroplasty” OR “total hip replacement”; third search string: “osteotomy” OR “graft” OR “rod” OR “fusion” OR “arthrodesis”. Truncated search terms utilizing the wildcard character and the “related articles” function were used to broaden the search. Additionally, the references of included articles were hand-searched to identify any additional studies.

## Study selection

All observational studies that compared outcomes following THA in individuals with and without prior surgery of the femur or pelvis were included. The following inclusion criteria were applied: (a) adult population with or without a history of surgery to the femur or pelvis in childhood; (b) THA in adulthood; and (c) compared outcomes following THA. However, studies that included individuals in whom the index surgery was due to trauma were excluded. The following additional exclusion criteria were applied: (a) non-English language; (b) non-human studies; (c) experimental trials; (d) review articles; (e) editorials; (f) case reports; (g) case series; (h) letters; (i) conference abstracts; and (j) unpublished studies.

## Outcome measures

The following outcome measures were evaluated: (a) operative time; (b) blood loss; (c) intra- and post-operative complications for which we pragmatically accepted the definitions provided by the authors; (d) implant survivorship as defined by the time from surgery to revision; (e) radiological outcome as defined by component positioning, wear or loosening; and (f) functional outcome as defined by validated outcome scores (e.g. Harris Hip Score, Oxford Hip Score). Studies that reported at least one of the outcomes of interest were included in the meta-analysis. The primary outcome was implant survivorship as this was considered to be the most reliable and consistent measurement.

## Data extraction

Two independent reviewers (E.L.G and O.R.B.) screened all the titles and abstracts for inclusion, both of whom were blinded to authors, journals, institutional affiliations and dates of publication. Both reviewers evaluated each selected reference independently and summarized relevant study characteristics. In case of disagreement, a consensual decision between the two reviewers under the involvement of a third independent reviewer (T.D.) was reached. The following data items were extracted: the year of publication, study design, sample size, type of patients, patient characteristics and outcome measures.

## Quality and risk of bias assessment

The Methodological Index for Non-Randomized Studies (MINORS) was used for quality and risk of bias assessment [6]. The risk of publication bias was assessed using a funnel plot. Assessments of publication bias for each study design were performed separately. The quality of evidence was assessed as per GRADE. Quality assessment was carried out by two independent reviewers.

## Statistical analysis

Risk ratio (RR), mean difference (MD) and hazard ratio (HR) are presented with 95% confidence intervals (CI). HR was used as a summary statistic for long-term outcomes (survival analysis) as described by Parmar et al. [7]. Review Manager 5.4 (Cochrane Collaboration, Oxford, United Kingdom) was used for data analysis. Medians were converted to means using the formula described by Hozo et al. [8]. The fixed-effects model was used to pool the results. The standard heterogeneity test, the *I*^2^ statistic, was used to assess the consistency of the effect sizes, which indicates the percentage of the variability in effect estimates because of true between-study variance rather than within-study variance. Statistical heterogeneity was defined as low, moderate and high with an *I*^2^ of above 25%, 50% and 75%, respectively [9]. Results above 60% were considered as substantial heterogeneity. Subgroup analyses based on the type of prior surgery (e.g. femoral osteotomy, pelvic osteotomy) performed were conducted.

## Results

### Study characteristics

**Figure 1:**
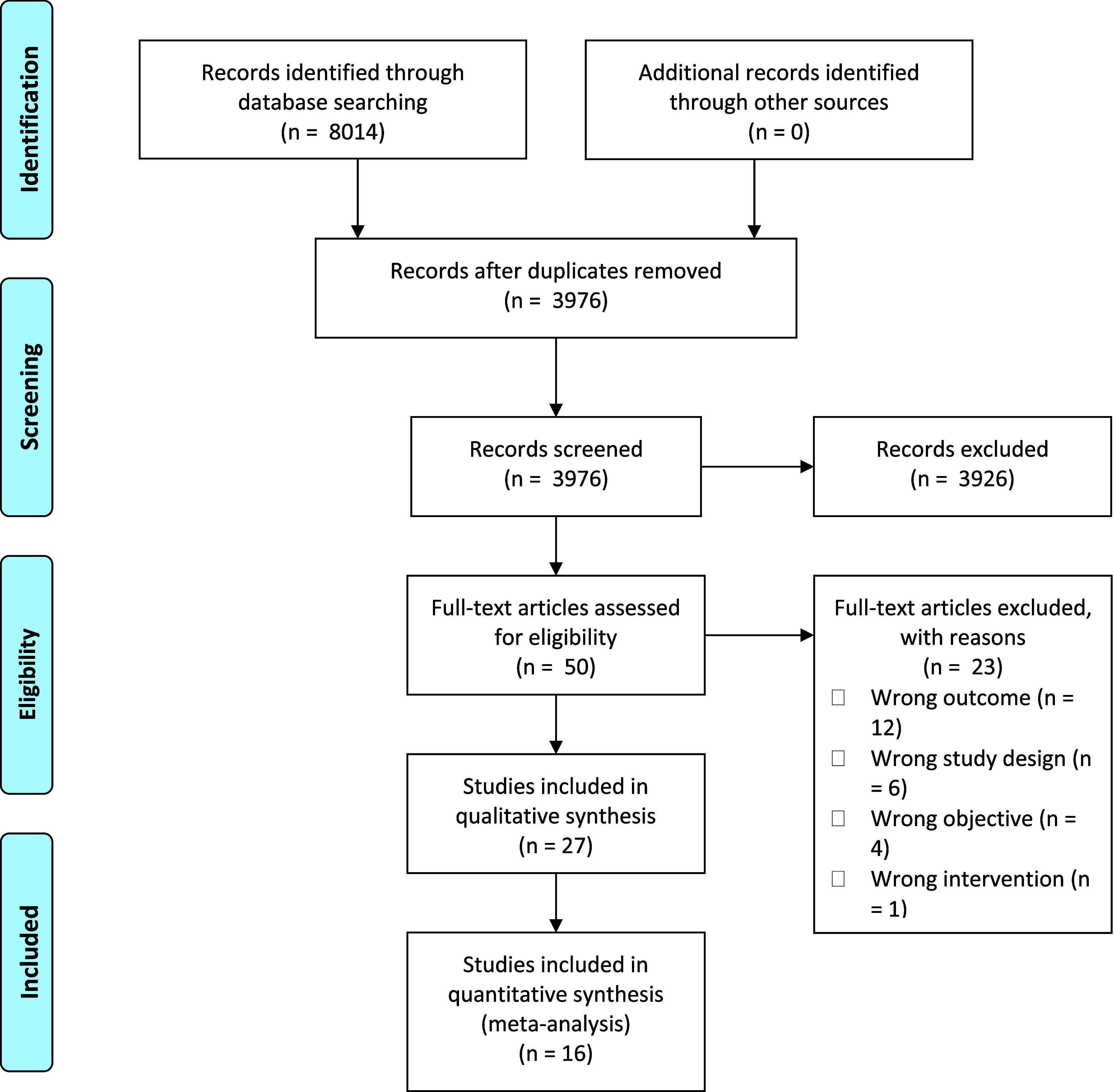
Study flowchart showing screening and selection process.

A total of 16 studies comprising 2576 patients were included, which compared 939 patients with prior joint-preserving surgery (PS) with 1637 patients with no prior joint-preserving surgery (NPS), (Tables 1, 2 and Figure 1) [10–25].

**Table 1:** Study characteristics.

Study	Years	Study design	Comparison	Sample size	Hips	Follow-up (years)
Amanatullah et al.	2015	Retrospective cohort (comparative)	Matched cohort of THAs without prior periacetabular osteotomy	45 (22, 23)	46 (23, 23)	10 (4), 6 (4)
Boos et al.	1997	Retrospective cohort (comparative)	Matched cohort of THAs without prior proximal femoral osteotomy	148 (74, 74)	148 (74, 74)	82.6 (57–116)*
Erdoğan and Can	2020	Retrospective cohort (comparative)	Cohort of THAs without prior pelvic or proximal femoral osteotomies	260 (88, 172)	358 (118, 240)	41 (24–76)*, 43.2 (24–74)*
George et al.	2018	Retrospective cohort (comparative)	Cohort of THAs without prior hip salvage procedure	174 (34, 140)	215 (37, 178)	2
Hashemi-Nejad et al.	2002	Retrospective cohort (comparative)	Matched cohort of THAs without prior Chiari osteotomy	72 (26, 46)	78 (28, 50)	60 (25–199)*
Haverkamp et al.	2006	Retrospective cohort (comparative)	Matched cohort of THAs without prior intertrochanteric osteotomy	361 (108, 253)	411 (121, 290)	19.2 (11.3–29)
Kawasaki et al.	2005	Retrospective cohort (comparative)	Matched cohort of THAs without prior transtrochanteric rotational osteotomy	29 (14, 15)	31 (15, 16)	5.0 (3.4–8.7), 4.9 (3.0–6.6)
Lee et al.	2009	Retrospective cohort (comparative)	Matched cohort of THAs without prior transtrochanteric rotational osteotomy	37 (13, 24)	42 (14, 28)	4.8 (2.7), 5.1 (2.7)
Migaud et al.	2014	Case-control	Matched control group of THAs without prior bone surgery	332 (128, 204)	430 (159, 271)	13.2 (5.4)
Minoda et al.	2006	Retrospective cohort (comparative)	Matched cohort of THAs without prior Chiari osteotomy	25 (8, 17)	30 (10, 20)	2.7 (2–5)*, 3.1 (2–5)*
Osawa et al.	2016	Case-control	Matched control group of THAs without prior periacetabular osteotomy	144 (48, 96)	156 (52, 104)	5.4 (3.2), 5.3 (3.1)
Peters et al.	2001	Retrospective cohort (comparative)	Matched cohort of THAs without prior triple innominate osteotomy	22 (11, 11)	26 (13, 13)	36, 28
Richards et al.	2011	Retrospective cohort (comparative)	Matched cohort of primary and revision THAs	77 (26, 34, 17)	77 (26, 34, 17)	9 (2–21)
Søballe et al.	1989	Retrospective cohort (comparative)	Cohort of THAs without prior osteotomy	359 (116, 243)	392 (116, 276)	56, 54
Tamaki et al.	2016	Retrospective cohort (comparative)	Cohort of THAs with prior Chiari osteotomy vs rotational periacetabular osteotomy vs shelf acetabuloplasty compared to cohort of primary THAs	49 (13, 21, 15)	51 (13, 22, 16)	3.5 (2–7)*, 3.8 (2–7)*, 4.0 (2–6)*
Tokunaga et al.	2011	Retrospective cohort (comparative)	Control group of THAs without prior Salter or Chiari osteotomies	87 (45, 42)	103 (52, 51)	7.3 (3–15)*, 8.5 (3–17)*

**Table 2:** Patient characteristics.

Study	Age	Age at index procedure	Laterality (R, L, B)	M, F	Etiology	Procedure	Time from index procedure to THA (years)
Amanatullah et al.	38 (11), 38 (10)	–	–	11, 34	Developmental dysplasia of the hip (45)	Periacetabular osteotomy	5 (1–10)
Boos et al.	57.4 (34–79), 61.6 (33–82)	–	–	74, 74	Osteoarthritis (39, 39), congenital hip dislocation (25, 25), avascular necrosis (4, 4), other (6, 6)	Proximal femoral osteotomy	10.67 (1–20)
Erdoğan and Can	40.59 (23–82)*, 46.88 (23–82)*	–	–	22, 238	Developmental dysplasia of the hip (260)	Salter osteotomy (38), Chiari osteotomy (36), Pemberton osteotomy (22), shelf acetabuloplasty (5), triple innominate osteotomy (2), Schanz osteotomy (15), varus-derotation (5)	29.5 (3–53)
George et al.	23.1 (4.9), 23.9 (4.5)	–		91, 124	Avascular necrosis (17, 102), acetabular dysplasia (10, 30), other (10, 46)	Multiple	4.9 (5.4)
Hashemi-Nejad et al.	45 (38–64), 39 (25–51)	–	27, 39, 6	6, 66	Developmental dysplasia of the hip (72)	Chiari osteotomy	17 (4–28)
Haverkamp et al.	–	–	–	–	–	Intertrochanteric osteotomy	–
Kawasaki et al.	43 (30–53), 47 (21–57)	39 (20–51)	–	23, 6	Avascular necrosis (14)	Transtrochanteric rotational osteotomy	4 (0.5–7)
Lee et al.	40.0 (8.8), 40.1 (8.8)	38.3 (23–49)	–	28, 9	Osteonecrosis (13)	Transtrochanteric anterior rotational osteotomy	1.7 (0.3–3.1)
Migaud et al.	55 (17–75), 57 (18–80)	–	–	63, 269	Congenital hip dislocation (128)	Hip shelf (46), Chiari osteotomy (16), Salter osteotomy (2), Milch osteotomy (36), other femoral osteotomy (45), shelf + Milch osteotomy (1), shelf + femoral osteotomy (4), Chiari + femoral osteotomy (6), Salter + femoral osteotomy (1), Chiari + shelf (1), femoral osteotomy + arthrodesis (1)	–
Minoda et al.	55.4 (7.2), 56.3 (4.6)	–	–	0, 25	Developmental dysplasia of the hip (25)	Chiari osteotomy	–
Osawa et al.	56.5 (6.4), 57.0 (6.3)	–	–	4, 140	Developmental dysplasia of the hip (144)	Periacetabular osteotomy	11.4 (6.8)
Peters et al.	37 (16–50), 41 (17–54)	31 (13–43)	–	4, 18	Acetabular dysplasia (11)	Triple inominate osteotomy	–
Richards et al.	49 (25–74), 67*	29 (7–46)	–	–	–	Arthrodesis	–
Søballe et al.	63 (30–80)*, 64 (23–94)*	–	–	197, 162	Osteoarthritis, congenital hip dislocation	Intertrochanteric osteotomy	–
Tamaki et al.	57.5 (44–70)*, 56.8 (34–73)*, 54.6 (41–78)*	35.1 (20–49)*, 36.1 (16–59)*, 26.8 (17–47)*	–	4, 45	Acetabular dysplasia (49)	Chiari osteotomy (13), rotational periacetabular osteotomy (21), shelf acetabuloplasty (16)	22.4 (4–39)*, 20.7 (8–35)*, 28.1 (13–43)*
Tokunaga et al.	41 (25–58)*, 47 (27–66)*	–	–	4, 83	Developmental dysplasia of the hip (87)	Salter osteotomy (40), Chiari osteotomy (9), Chiari + Salter (3)	19.3 (1–40)

### Quality assessment and risk of bias

**Figure 2:**
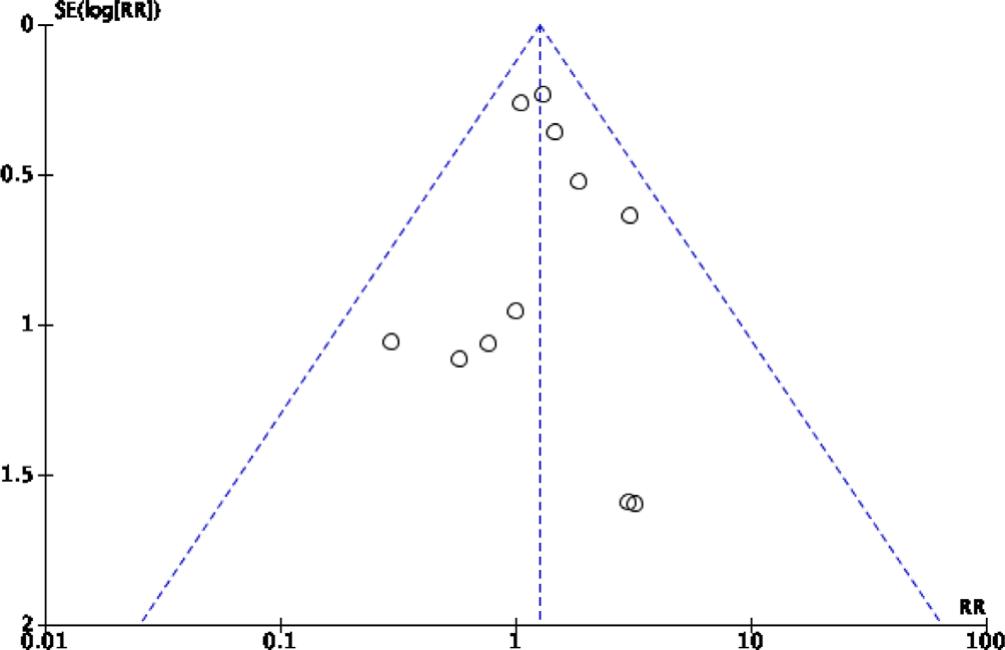
Funnel plot assessing publication bias for the outcome, revision surgery.

A maximum score of 24 points was used for all comparative studies. The median MINORS score was 18 points, with a range of 15–21 points. Overall, the studies included scored well in terms of the presence of a clearly stated aim, use of endpoints appropriate to the study, use of an unbiased assessment of the study endpoints, an adequate control group, and adequate statistical analyses (Table 3). However, the studies included scored poorly in terms of performing a prospective calculation of the study size, inclusion of consecutive patients, and prospective collection of data. Publication bias was assessed with the use of a funnel plot and presented for the outcome, revision surgery (Figure 2). The overall risk of publication bias was deemed to be low.

**Table 3:** MINORS quality and risk of bias assessment.

Study	Total	A clearly stated aim	Inclusion of consecutive patients	Prospective collection of data	Endpoints appropriate to the aim of the study	Unbiased assessment of the study endpoint	Follow-up period appropriate to the aim of the study	Loss to follow up less than 5%	Prospective calculation of the study size	An adequate control group	Contemporary groups	Baseline equivalence of groups	Adequate statistical analyses
Amanatullah et al.	18	2	0	1	2	2	2	2	0	2	1	2	2
Boos et al.	17	2	0	1	2	2	2	1	0	2	2	1	2
Erdoğan and Can	18	2	0	1	2	2	2	2	0	2	2	1	2
George et al.	18	2	2	1	2	2	2	1	0	2	0	2	2
Hashemi-Nejad et al.	19	2	0	1	2	2	2	2	0	2	2	2	2
Haverkamp et al.	20	2	2	1	2	2	2	2	0	2	2	1	2
Kawasaki et al.	20	2	2	1	2	2	2	1	0	2	2	2	2
Lee et al.	15	2	0	1	2	2	2	0	0	2	0	2	2
Migaud et al.	19	2	2	1	2	2	1	1	0	2	2	2	2
Minoda et al.	16	2	0	0	2	2	2	0	0	2	2	2	2
Osawa et al.	17	2	2	0	2	2	1	0	0	2	2	2	2
Peters et al.	17	2	0	1	2	2	2	2	0	2	0	2	2
Richards et al.	16	2	0	1	2	2	2	2	0	2	0	1	2
Søballe et al.	19	2	2	0	2	2	2	2	0	2	2	1	2
Tamaki et al.	21	2	2	1	2	2	2	2	0	2	2	2	2
Tokunaga et al.	16	2	0	1	2	2	2	2	0	2	0	1	2

### Intra-operative outcomes

**Figure 3:**
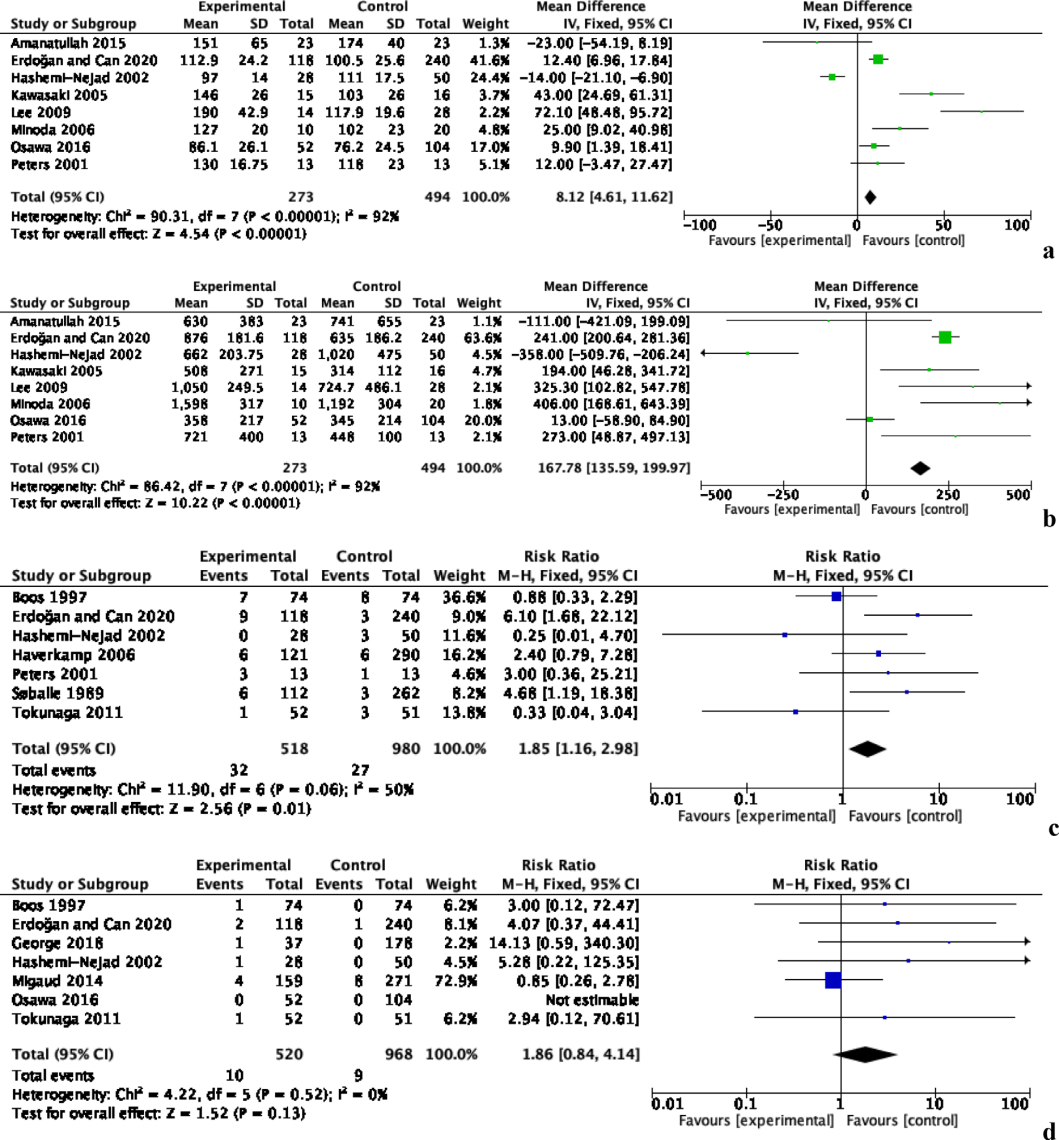
Intra-operative outcomes of total hip arthroplasty with and without prior bone surgery. (a) Operating time, (b) blood loss, (c) peri-prosthetic fracture and (d) nerve injury**.**

Intra-operative outcomes including operating time, blood loss, peri-prosthetic fracture, and nerve injury were reported in 13 studies, comprising 2018 patients (Figure 3). Eight studies with 767 patients reported data on operating time, which was significantly longer in the PS group by an MD of 8.12 [95% CI: 4.61, 11.62, *p* < 0.00001, *I*^2^ = 92%] (Figure 3a). Subgroup analyses demonstrated that this significant difference was attributed to prior femoral osteotomy. There was significant heterogeneity in the data and the evidence was deemed to be of low quality.

Blood loss was reported by eight studies of 767 patients (Figure 3b). There was significantly greater blood loss in the PS group, with an MD of 167.87 [95% CI: 135.59, 199.97, *p* < 0.00001, *I*^2^ = 92%]. Subgroup analyses revealed that this significant difference was due to prior femoral osteotomy. Significant heterogeneity was present in the data and the evidence was deemed to be of low quality.

The incidence of peri-prosthetic fracture was reported by seven studies with 1498 patients, which was significantly higher in the PS group, with an RR of 1.85 [95% CI: 1.16, 2.98, *p* = 0.01, *I*^2^ = 50%] (Figure 3c). Subgroup analyses demonstrated that this significant difference was attributed to prior femoral osteotomy. There was moderate heterogeneity in the data and the evidence was deemed to be of moderate quality.

Seven studies comprising 1488 patients reported whether nerve injury was present (Figure 3d). There was no significant difference between the PS and NPS groups, with an RR of 1.85 [95% CI: 0.84, 1.14, *p* = 0.13, *I*^2^ = 0%]. Subgroup analyses did not identify any procedures that were associated with an increased risk of nerve injury. No heterogeneity was present in the data and the evidence was deemed to be of moderate quality.

### Post-operative clinical outcomes

**Figure 4:**
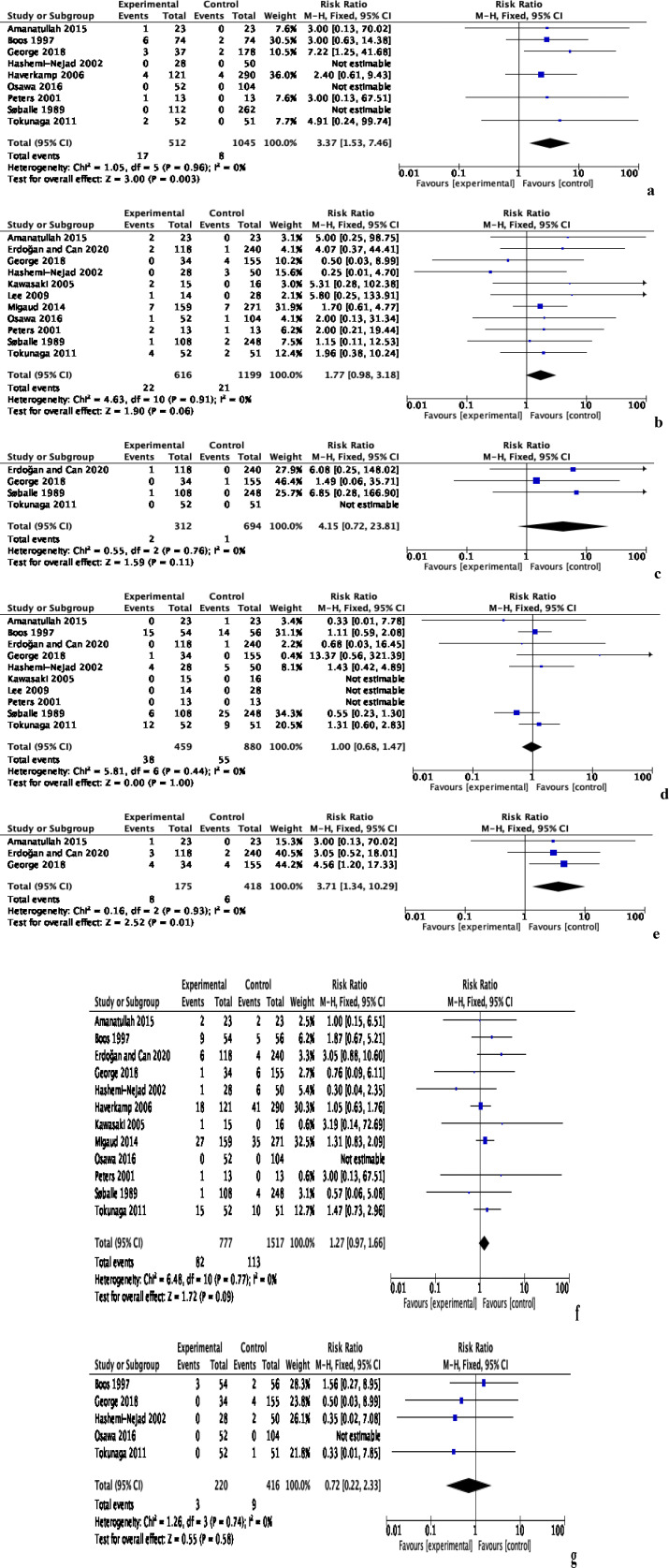
Post-operative outcomes of total hip arthroplasty with and without prior bone surgery. (a) Surgical site infection, (b) dislocation, (c) peri-prosthetic fracture, (d) loosening, (e) re-operation, (f) revision and (g) venous thromboembolism.

Post-operative clinical outcomes including surgical site infection (SSI), dislocation, peri-prosthetic fracture, aseptic loosening, revision surgery, and venous thromboembolism (VTE) were reported by 13 studies comprising 2429 patients (Figure 4). The incidence of SSI was reported by nine studies of 1557 patients, and was significantly higher in the PS group by an RR of 3.37 [95% CI: 1.53, 7.46, *p* = 0.003, *I*^2^ = 0%] (Figure 4a). Subgroup analyses did not identify differences in risk of SSI between different procedures. There was no heterogeneity in the data and the evidence was deemed to be of moderate quality.

The risk of dislocation was reported in 11 studies comprising 1815 patients (Figure 4b). There was no significant difference between the PS and NPS groups, with an RR of 1.77 [95% CI: 0.98, 3.18, *p* = 0.06, *I*^2^ = 0%]. Subgroup analyses did not identify any procedures that were associated with an increased risk of dislocation. No heterogeneity was present in the data and the evidence was deemed to be of moderate quality.

Four studies with 1006 patients reported the occurrence of peri-prosthetic fracture (Figure 4c). No significant difference was observed between the two groups, with an RR of 4.15 [95% CI: 0.72, 23.81, *p* = 0.11, *I*^2^ = 0%]. Subgroup analyses did not identify differences in the risk of peri-prosthetic fracture between different procedures. There was no heterogeneity in the data and the evidence was deemed to be of low quality.

The incidence of aseptic loosening was reported in 10 studies comprising 1339 patients, which showed comparable risk between the PS and NPS groups, with an RR of 1.00 [95% CI: 0.68, 1.47, *p* = 1.00, *I*^2^ = 0%] (Figure 4d). Subgroup analyses did not identify any procedures that were associated with an increased risk of aseptic loosening. No heterogeneity was present in the data and the evidence was deemed to be of moderate quality.

Overall, 12 studies with 2296 patients reported the risk of revision surgery (Figure 4e). There was no significant difference between the two groups, with an RR of 1.27 [95% CI: 0.97, 1.66, *p* = 0.09, *I*^2^ = 0%]. Subgroup analyses did not identify any procedures that were associated with an increased risk of revision surgery. There was no heterogeneity in the data and the evidence was deemed to be of low quality.

Five studies with 636 patients documented the occurrence of venous thromboembolism (VTE) (Figure 4f). No significant difference was observed between the two groups, with a RR of 0.72 [95% CI: 0.22, 2.33, *p* = 0.58, *I*^2^ = 0%]. Subgroup analyses demonstrated comparable risk across all procedures and the evidence was deemed to be of low quality.

### Implant survivorship

**Figure 5:**
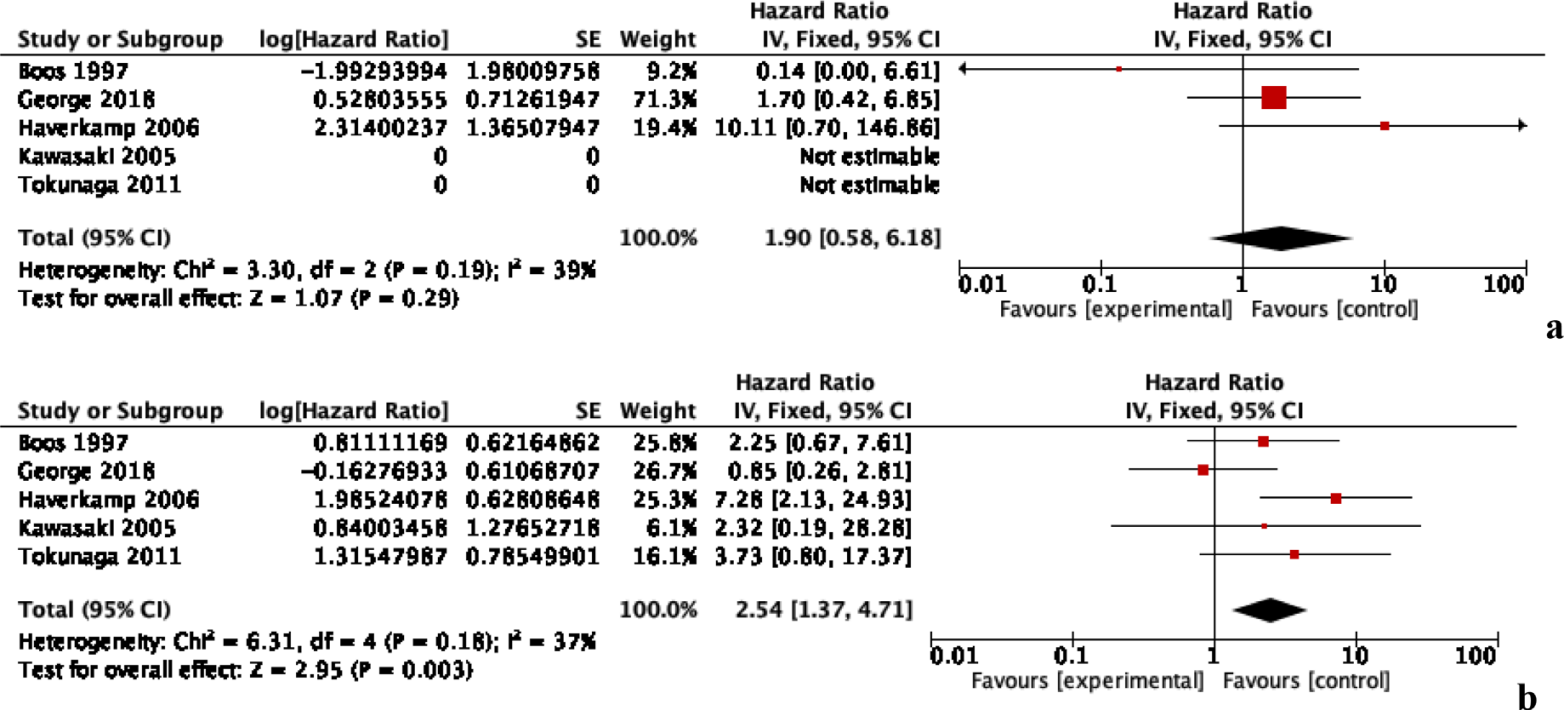
Implant survivorship with and without prior bone surgery. (a) One-year follow-up and (b) five years follow-up.

Data regarding implant survivorship was available from five studies of 885 patients, with at least five years of follow-up (Figure 5). At one-year, implant survivorship between the PS and NPS groups was comparable with an HR of 1.90 [95% CI: 0.58, 6.18, *p* = 0.29, *I*^2^ = 39%]. At five years, the PS group had significantly inferior implant survivorship with an HR of 2.54 [95% CI: 1.37, 4.71, *p* = 0.003, *I*^2^ = 37%]. Subgroup analyses observed this difference to be due to prior femoral osteotomy. Low heterogeneity was present in the data and the evidence was deemed to be of moderate quality.

### Functional outcome

**Figure 6:**
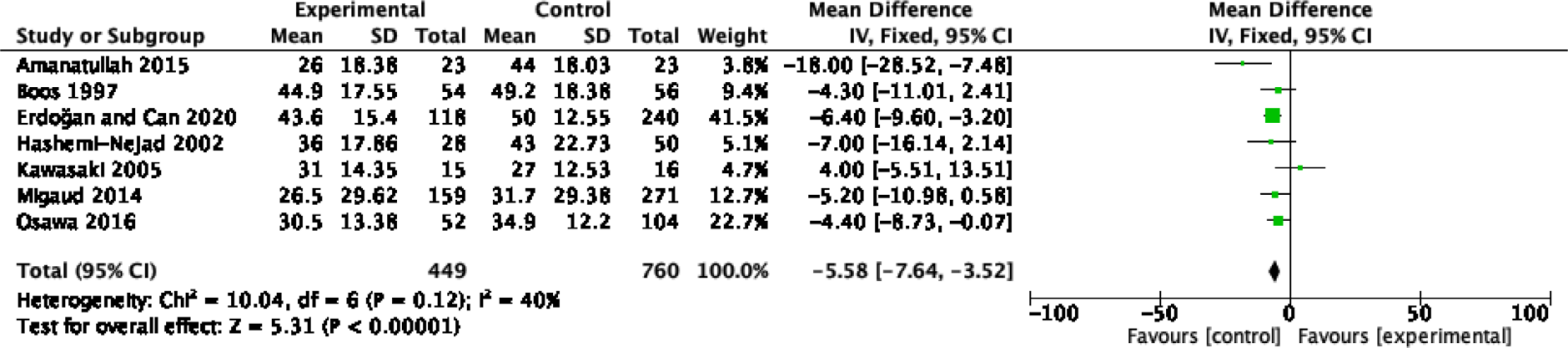
Functional outcome of total hip arthroplasty with and without prior bone surgery. (a) ΔHHS.

Seven studies, comprising 1209 patients, provided functional outcome scores in the form of the Harris Hip Score (HHS) (Figure 6). Compared to the NPS group, the PS group experienced a significantly poorer improvement in HHS with an MD of −5.58 [95% CI: −7.64, −3.52, *p* < 0.00001, *I*^2^ = 40%]. Subgroup analyses identified that this was attributed to prior acetabular osteotomy. Low heterogeneity was present in the data and the evidence was deemed to be of moderate quality.

## Discussion

The present meta-analysis reports intra-operative, post-operative clinical and functional outcomes, and implant survivorship in patients with and without previous joint-preserving hip procedures. Furthermore, this work builds on existing research on this topic by identifying procedures that have the potential to compromise outcomes following subsequent THA.

In this study, THA in patients with prior femoral osteotomy was associated with significantly longer operating time, greater blood loss, and elevated risk of peri-prosthetic fracture. Femoral osteotomy leads to distortion of the proximal femur, thus, making subsequent THA more technically challenging [14]. In particular, preparation of the femoral canal for the implant is affected by displacement of the femoral shaft, angulation of the femur or retained hardware [18]. Duncan et al. reported longer operating time and greater blood loss in the prior femoral osteotomy cohort, which corroborate our current findings [3].

Inferior clinical outcomes were initially observed in these patients undergoing prior joint-preserving hip procedures, although these studies lacked robustness and methodological rigour due to the lack of control and comparator groups [26, 27]. Subsequent systematic reviews reporting comparable clinical outcomes have challenged these observations [3, 4]. The risk of SSI is markedly increased in patients with any prior joint-preserving hip procedure, which is in line with previous data [14]. However, the risks of dislocation, peri-prosthetic fracture, aseptic loosening, and revision surgery do appear comparable.

Implant survivorship of THA following prior femoral osteotomy was significantly poorer at five years follow-up, which contrasts with previous work by Duncan et al. [3] and Gallazi et al. [4]. Nonetheless, evaluation of the data suggests that the difference in implant survivorship can be attributed to the higher risk of prosthetic joint infection (PJI) following THA in patients with prior femoral osteotomy. Prior reviews have also been limited by the inclusion of studies with small cohorts, significant heterogeneity and lack of imputable survivorship data, all of which increase the probability of a Type II error.

The functional improvement conferred by THA appears to be compromised by prior acetabular osteotomy. Acetabular osteotomy alters the anatomy and version of the acetabulum. Hence, retroversion of the acetabular component of the implant may occur, which could explain the smaller functional improvement following THA [28]. Our updated analysis includes new studies with larger cohorts, thus, addressing the inadequate power, common to prior reviews evaluating THA following prior acetabular osteotomy [3]. This is the first study to report this and highlights the need for further analysis of hip centre and version alteration at the time of the hip preservation surgery. As with femoral osteotomies, there is more than one type of acetabular procedure and these need further evaluation.

This meta-analysis highlights the technical challenges of THA in patients with prior femoral osteotomy; notably, the increased operating time, blood loss and risk of peri-prosthetic fracture, as well as poorer long-term implant survivorship. However, consideration must be given to the type of femoral osteotomy performed when using this information for clinical decision-making. It is unlikely that just the act of femoral osteotomy is responsible but more probably the type and location. The complications are related to additional procedures required to normalise hip morphology to accommodate an implant in THA. Thus, while osteotomies in a subtrochanteric location will deform the femur and increase problems, it is possible that this is not true of trochanteric osteotomies and intra-articular osteotomies. Further studies are required to examine this.

There are several limitations that should be taken into consideration in the present meta-analysis. The studies included are retrospective in nature and therefore, susceptible to inherent biases and confounding influences [29]. Additionally, survivorship data is reported inconsistently by different studies, so a degree of imputation is required in our analysis. There was insufficient information to perform subgroup analyses to evaluate how much of the true effect size could be attributed to the choice of implant as well.

## Conclusion

This analysis shows that caution should be exercised when considering femoral osteotomies in patients who may later require THA as there is an association with reduced implant survivorship and peri-prosthetic fracture. Surgical decision-making for these complex cases is best managed by a high-volume hip preservation surgeon in conjunction with an arthroplasty surgeon specializing in complex primary and revision surgery, whilst ensuring the patient and their family are fully informed.

## Data Availability

Data are available on request.

## Appendix

### Literature search strategy

*Ovid MEDLINE*

1. pelvi*.mp. [mp=title, abstract, original title, name of substance word, subject heading word, floating sub-heading word, keyword heading word, organism supplementary concept word, protocol supplementary concept word, rare disease supplementary concept word, unique identifier, synonyms]
2. hip*.mp. [mp=title, abstract, original title, name of substance word, subject heading word, floating sub-heading word, keyword heading word, organism supplementary concept word, protocol supplementary concept word, rare disease supplementary concept word, unique identifier, synonyms]
3. fem?r*.mp. [mp=title, abstract, original title, name of substance word, subject heading word, floating sub-heading word, keyword heading word, organism supplementary concept word, protocol supplementary concept word, rare disease supplementary concept word, unique identifier, synonyms]
4. 1 or 2 or 3
5. total hip arthroplasty.mp. [mp=title, abstract, original title, name of substance word, subject heading word, floating sub-heading word, keyword heading word, organism supplementary concept word, protocol supplementary concept word, rare disease supplementary concept word, unique identifier, synonyms]
6. total hip replacement.mp. [mp=title, abstract, original title, name of substance word, subject heading word, floating sub-heading word, keyword heading word, organism supplementary concept word, protocol supplementary concept word, rare disease supplementary concept word, unique identifier, synonyms]
7. 5 or 6
8. child*.mp. [mp=title, abstract, original title, name of substance word, subject heading word, floating sub-heading word, keyword heading word, organism supplementary concept word, protocol supplementary concept word, rare disease supplementary concept word, unique identifier, synonyms]
9. p*ediatric*.mp. [mp=title, abstract, original title, name of substance word, subject heading word, floating sub-heading word, keyword heading word, organism supplementary concept word, protocol supplementary concept word, rare disease supplementary concept word, unique identifier, synonyms]
10. adolescen*.mp. [mp=title, abstract, original title, name of substance word, subject heading word, floating sub-heading word, keyword heading word, organism supplementary concept word, protocol supplementary concept word, rare disease supplementary concept word, unique identifier, synonyms]
11. teenage*.mp. [mp=title, abstract, original title, name of substance word, subject heading word, floating sub-heading word, keyword heading word, organism supplementary concept word, protocol supplementary concept word, rare disease supplementary concept word, unique identifier, synonyms]
12. 8 or 9 or 10 or 11
13. osteotom*.mp. [mp=title, abstract, original title, name of substance word, subject heading word, floating sub-heading word, keyword heading word, organism supplementary concept word, protocol supplementary concept word, rare disease supplementary concept word, unique identifier, synonyms]
14. graft*.mp. [mp=title, abstract, original title, name of substance word, subject heading word, floating sub-heading word, keyword heading word, organism supplementary concept word, protocol supplementary concept word, rare disease supplementary concept word, unique identifier, synonyms]
15. rod*.mp. [mp=title, abstract, original title, name of substance word, subject heading word, floating sub-heading word, keyword heading word, organism supplementary concept word, protocol supplementary concept word, rare disease supplementary concept word, unique identifier, synonyms]
16. fusion*.mp. [mp=title, abstract, original title, name of substance word, subject heading word, floating sub-heading word, keyword heading word, organism supplementary concept word, protocol supplementary concept word, rare disease supplementary concept word, unique identifier, synonyms]
17. 13 or 14 or 15 or 16
18. (slipped adj2 femoral epiphysis).mp. [mp=title, abstract, original title, name of substance word, subject heading word, floating sub-heading word, keyword heading word, organism supplementary concept word, protocol supplementary concept word, rare disease supplementary concept word, unique identifier, synonyms]
19. SCFE.mp. [mp=title, abstract, original title, name of substance word, subject heading word, floating sub-heading word, keyword heading word, organism supplementary concept word, protocol supplementary concept word, rare disease supplementary concept word, unique identifier, synonyms]
20. SUFE.mp. [mp=title, abstract, original title, name of substance word, subject heading word, floating sub-heading word, keyword heading word, organism supplementary concept word, protocol supplementary concept word, rare disease supplementary concept word, unique identifier, synonyms]
21. (developmental dysplasia adj3 hip).mp. [mp=title, abstract, original title, name of substance word, subject heading word, floating sub-heading word, keyword heading word, organism supplementary concept word, protocol supplementary concept word, rare disease supplementary concept word, unique identifier, synonyms]
22. DDH.mp. [mp=title, abstract, original title, name of substance word, subject heading word, floating sub-heading word, keyword heading word, organism supplementary concept word, protocol supplementary concept word, rare disease supplementary concept word, unique identifier, synonyms]
23. 18 or 19 or 20 or 21 or 22
24. 4 and 7 and 12 and 17 and 23

*Ovid Embase*

1. pelvi*
2. hip
3. fem?r
4. 1 or 2 or 3
5. total hip arthroplasty
6. total hip replacement
7. 5 or 6
8. child
9. p*ediatric*
10. adolescen*
11. teenage*
12. 8 or 9 or 10 or 11
13. osteotom*
14. graft*
15. rod*
16. fusion*
17. 13 or 14 or 15 or 16
18. (slipped adj2 femoral epiphysis)
19. SCFE
20. SUFE
21. (developmental dysplasia adj3 hip)
22. DDH
23. 18 or 19 or 20 or 21 or 22
24. 4 and 7 and 12 and 17 and 23

*Scopus*

1. pelvi* OR hip OR fem?r
2. total hip arthroplasty OR total hip replacement
3. child OR p*ediatric* OR adolescen* OR teenage*
4. osteotom* OR graft* OR rod* OR fusion*
5. slipped capital femoral epiphysis OR SCFE OR slipped upper femoral epiphysis OR SUFE OR developmental dysplasia hip OR DDH
6. 1 and 2 and 3 and 4 and 5

## References

[1] Leunig M, Ganz R (2014) The evolution and concepts of joint-preserving surgery of the hip. Bone Joint J 96-B(1): 5–18. 10.1302/0301-620X.96B1.32823. 24395304

[2] Adler KL, Cook PC, Yen Y-M, Giordano BD (2015) Current concepts in hip preservation surgery: Part I. Sports Health 7(6), 518–526. 10.1177/1941738115587270. 26502445 PMC4622374

[3] Duncan S, Wingerter S, Keith A, Fowler SA, Clohisy J (2015) Does previous osteotomy compromise total hip arthroplasty? A systematic review. J Arthroplasty 30(1), 79–85. 10.1016/j.arth.2014.08.030. 25262440

[4] Gallazzi E, Morelli I, Peretti G, Zagra L (2019) What is the impact of a previous femoral osteotomy on THA? A systematic review. Clin Orthop Relat Res 477(5), 1176–1187. 10.1097/CORR.0000000000000659. 30998636 PMC6494317

[5] Stroup DF, Berlin JA, Morton SC, et al. (2000) Meta-analysis of observational studies in epidemiology: A proposal for reporting. Meta-analysis Of Observational Studies in Epidemiology (MOOSE) group. JAMA 283(15), 2008–2012. 10.1001/jama.283.15.2008. 10789670

[6] Slim K, Nini E, Forestier D, Kwiatkowski F, Panis Y, Chipponi J (2003) Methodological index for non-randomized studies (Minors): Development and validation of a new instrument. ANZ J Surg 73(9), 712–716. 10.1046/j.1445-2197.2003.02748.x. 12956787

[7] Parmar MK, Torri V, Stewart L (1998) Extracting summary statistics to perform meta analyses of the published literature for survival endpoints. Stat Med 17(24), 2815–2834. 10.1002/(sici)1097-0258(19981230)17:24<2815::aid373sim110>3.0.co;2-8. 9921604

[8] Hozo SP, Djulbegovic B, Hozo I (2005) Estimating the mean and variance from the median, range, and the size of a sample. BMC Med Res Methodol 5, 13. 10.1186/1471-2288-5-13. 15840177 PMC1097734

[9] Higgins JP, Thompson SG, Deeks JJ, Altman DG (2003) Measuring inconsistency in meta-analyses. Br Med J 327(7414), 557–560. 10.1136/bmj.327.7414.557. 12958120 PMC192859

[10] Tamaki T, Oinuma K, Miura Y, Shiratsuchi H (2016) Total hip arthroplasty after previous acetabular osteotomy: Comparison of three types of acetabular osteotomy. J Arthroplasty 31(1), 172–175. 10.1016/j.arth.2015.07.018. 26264177

[11] George J, Miller EM, Higuera CA, Kuivila TE, Mont MA, Goodwin RC (2018) Influence of prior hip salvage surgery on outcomes after total hip arthroplasty in young patients. J Arthroplasty 33(4), 1108–1112. 10.1016/j.arth.2017.11.008. 29198874

[12] Erdoğan F, Can A (2020) The effect of previous pelvic or proximal femoral osteotomy on the outcomes of total hip arthroplasty in patients with dysplastic coxarthrosis. Acta Orthop Traumatol Turc 54(1), 74–82. 10.5152/j.aott.2020.01.7. 32175900 PMC7243695

[13] Søballe K, Boll KL, Kofod S, Severinsen B, Kristensen SS (1989) Total hip replacement after medial-displacement osteotomy of the proximal part of the femur. J Bone Joint Surg Am 71(5), 692–697. 2732258

[14] Boos N, Krushell R, Ganz R, Müller ME (1997) Total hip arthroplasty after previous proximal femoral osteotomy. J Bone Joint Surg Br 79(2), 247–253. 10.1302/0301-620x.79b2.6982. 9119851

[15] Peters CL, Beck M, Dunn HK (2001) Total hip arthroplasty in young adults after failed triple innominate osteotomy. J Arthroplasty 16(2), 188–195. 10.1054/arth.2001.20903. 11222892

[16] Hashemi-Nejad A, Haddad FS, Tong KM, Muirhead-Allwood SK, Catterall A (2002) Does Chiari osteotomy compromise subsequent total hip arthroplasty? J Arthroplasty 17(6), 731–739. 10.1054/arth.2002.31974. 12216027

[17] Kawasaki M, Hasegawa Y, Sakano S, Masui T, Ishiguro N (2005) Total hip arthroplasty after failed transtrochanteric rotational osteotomy for avascular necrosis of the femoral head. J Arthroplasty 20(5), 574–579. 10.1016/j.arth.2005.01.018. 16309991

[18] Haverkamp D, de Jong PT, Marti RK (2006) Intertrochanteric osteotomies do not impair long-term outcome of subsequent cemented total hip arthroplasties. Clin Orthop Relat Res 444, 154–160. 10.1097/01.blo.0000194066.10227.1e. 16523138

[19] Minoda Y, Kadowaki T, Kim M (2006) Total hip arthroplasty of dysplastic hip after previous Chiari pelvic osteotomy. Arch Orthop Trauma Surg 126(6), 394–400. 10.1007/s00402-006-0141-6. 16628429

[20] Lee YK, Ha YC, Kim KC, Yoo JJ, Koo KH (2009) Total hip arthroplasty after previous transtrochanteric anterior rotational osteotomy for femoral head osteonecrosis. J Arthroplasty 24(8), 1205–1209. 10.1016/j.arth.2009.04.013. 19523785

[21] Richards CJ, Duncan CP (2011) Conversion of hip arthrodesis to total hip arthroplasty: Survivorship and clinical outcome. J Arthroplasty 26(3), 409–413. 10.1016/j.arth.2010.02.005. 20346614

[22] Tokunaga K, Aslam N, Zdero R, Schemitsch EH, Waddell JP (2011) Effect of prior salter or chiari osteotomy on THA with developmental hip dysplasia. Clin Orthop Relat Res 469(1), 237–243. 10.1007/s11999-010-1375-8. 20458643 PMC3008866

[23] Migaud H, Putman S, Berton C, et al. (2014) Does prior conservative surgery affect survivorship and functional outcome in total hip arthroplasty for congenital dislocation of the hip? A case-control study in 159 hips. Orthop Traumatol Surg Res 100(7), 733–737. 10.1016/j.otsr.2014.07.016. 25281551

[24] Amanatullah DF, Stryker L, Schoenecker P, et al. (2015) Similar clinical outcomes for THAs with and without prior periacetabular osteotomy. Clin Orthop Relat Res 473(2), 685–691. 10.1007/s11999-014-4026-7. 25359629 PMC4294924

[25] Osawa Y, Hasegawa Y, Seki T, Amano T, Higuchi Y, Ishiguro N (2016) Significantly poor outcomes of total hip arthroplasty after failed periacetabular osteotomy. J Arthroplasty 31(9), 1904–1909. 10.1016/j.arth.2016.02.056. 27036922

[26] Iwase T, Hasegawa Y, Iwasada S, Kitamura S, Iwata H (1999) Total hip arthroplasty after failed intertrochanteric valgus osteotomy for advanced osteoarthrosis. Clin Orthop Relat Res 364, 175–181. 10.1097/00003086-199907000-00023. 10416407

[27] Suominen S, Antti-Poika I, Santavirta S, Konttinen YT, Honkanen V, Lindholm TS (1991) Total hip replacement after intertrochanteric osteotomy. Orthopedics 14(3), 253–257. 2020624

[28] Parvizi J, Burmeister H, Ganz R (2004) Previous Bernese periacetabular osteotomy does not compromise the results of total hip arthroplasty. Clin Orthop Relat Res 423, 118–122. 10.1097/01.blo.0000128287.98083.63. 15232436

[29] Metelli S, Chaimani A (2020) Challenges in meta-analyses with observational studies. Evid Based Ment Health 23(2), 83–87. 32139442 10.1136/ebmental-2019-300129 PMC10231593

